# Hydroquinidine Modulates Histopathological, Inflammatory, Apoptotic, EMT-Related, and PI3K/AKT/mTOR-Associated Markers in a DMH-Induced Rat Model of Colon Cancer

**DOI:** 10.3390/ijms27135672

**Published:** 2026-06-23

**Authors:** İlknur Keskin, Begüm Şahin, Aziz Bülbül, Mustafa Çakır, Mervenur Yavuz, Muhammet Volkan Bülbül, Turan Demircan

**Affiliations:** 1 Histology and Embryology Department, School of Medicine, İstanbul Medipol University, İstanbul 34810, Turkey; 2 Program of Histology and Embryology, Institute of Health Sciences, İstanbul Medipol University, İstanbul 34810, Turkey; begum.sahin@medipol.edu.tr; 3 Department of Physiology, Faculty of Milas Veterinary Medicine, Muğla Sıtkı Kocman University, Muğla 48200, Turkey; azizbulbul@mu.edu.tr; 4 Veterinary Physiology Program, Institute of Health Sciences, Muğla Sıtkı Kocman University, Muğla 48200, Turkey; mustafaccakir48@gmail.com; 5 Molecular Biology and Genetics Program, Institute of Natural Sciences, Muğla Sıtkı Kocman University, Muğla 48000, Turkey; ymervenuryavuz@gmail.com; 6 Histology and Embryology Department, School of Medicine, Ağrı İbrahim Çeçen University, Ağrı 04000, Turkey; mvbulbul@agri.edu.tr; 7 Medical Biology Department, School of Medicine, İzmir Bakircay University, İzmir 35665, Turkey

**Keywords:** Hydroquinidine, DMH-induced colon cancer, drug repurposing, pre-clinical safety, epithelial–mesenchymal transition, PI3K/AKT/mTOR pathway

## Abstract

Colon cancer remains a leading cause of cancer-related deaths, and drug repurposing offers a promising strategy to identify new therapies. Hydroquinidine (HQ), a class I antiarrhythmic agent, has recently been suggested to possess anticancer properties; however, its preclinical safety and efficacy in colorectal cancer are not well defined. The safety of HQ was evaluated in Wistar rats following OECD guidelines. Rats received daily intraperitoneal doses (2.5–25 mg/kg) for 90 days, with hematological, biochemical, and histopathological assessments performed. HQ was well tolerated up to 12.5 mg/kg, whereas 25 mg/kg caused signs of hepatotoxicity without lethality. A 1,2-dimethylhydrazine-induced colorectal cancer model was then used to assess HQ at safe doses (6.25 and 12.5 mg/kg) compared with cisplatin. Tissue histopathology and selected molecular markers associated with inflammation, apoptosis, epithelial–mesenchymal transition, and PI3K/AKT/mTOR pathway activity were analyzed. In the DMH-induced colon cancer model, HQ improved colonic tissue architecture and was associated with lower histopathological scores compared with untreated tumor controls. HQ also modulated tumor-associated markers by reducing IL-6 immunoreactivity, increasing caspase-3 expression, enhancing E-cadherin immunoreactivity, and decreasing vimentin expression. Moreover, HQ was associated with reduced immunoreactivity of mTOR pathway-related markers, suggesting attenuation of pathway activation in this experimental context. Overall, HQ showed an acceptable safety profile at the selected doses and exerted favorable histopathological and molecular modulatory effects, supporting further investigation as a potential repurposing candidate.

## 1. Introduction

Colorectal cancer remains one of the leading causes of cancer-related morbidity and mortality worldwide [1]. Despite advances in surgery, chemotherapy, and targeted therapies, having improved outcomes in early-stage disease [2,3], treatment of advanced stages of cancer is still limited by treatment resistance, recurrence, and systemic toxicity. These limitations highlight the pressing need for safer and more effective therapeutic strategies. In this context, drug repurposing has emerged as a promising approach to accelerate the development of novel anticancer agents by leveraging the established safety profiles of existing drugs [4].

Hydroquinidine (HQ), a stereoisomer of quinidine and a well-established class I antiarrhythmic drug, has traditionally been employed in the management of cardiac arrhythmias [5]. Beyond its cardiac applications, HQ exerts diverse pharmacological effects through its ability to block voltage-gated ion channels, including sodium and potassium channels [6]. Ion channels are increasingly recognized as critical regulators of cellular processes such as proliferation, apoptosis, migration, and invasion, all of which are dysregulated in cancer [7]. In particular, potassium channels such as hERG1 (Kv11.1), which is frequently overexpressed in colorectal tumors, have been implicated in promoting a proliferative and mesenchymal phenotype [8,9]. Pharmacological blockade of these channels has been shown to suppress tumor growth in several models, suggesting that antiarrhythmic agents like HQ may possess untapped anticancer potential.

Indeed, recent studies have begun to explore HQ’s activity against cancer cells. In vitro experiments have demonstrated that HQ exerts profound cytotoxic and antiproliferative effects across a spectrum of malignancies. In breast and ovarian cancer models, HQ markedly reduced cell viability, inhibited clonogenic and anchorage-independent growth, impaired migration, and induced apoptosis [10,11,12]. Proteomic analyses revealed widespread downregulation of cell-cycle-related proteins, including cyclin-dependent kinases and mini-chromosome maintenance proteins, along with activation of apoptosis and ferroptosis pathways [11]. Similar outcomes have been observed in non-small cell lung cancer cells, where HQ suppressed tumorigenic growth in spheroid models, inhibited migration, and triggered apoptotic cell death [13]. Most notably, in colon carcinoma cells, HQ treatment led to a pronounced reduction in colony-forming ability, migration, and proliferation, accompanied by stimulation of cell death and transcriptional changes favoring cell-cycle arrest and apoptosis [12]. These findings provide direct evidence that HQ can interfere with the growth and survival of colorectal cancer cells, establishing a rationale for its further evaluation in preclinical models.

Mechanistic insights support the view that HQ’s anticancer effects are multifaceted. By blocking potassium channels, HQ disrupts cellular membrane potential and ionic homeostasis, conditions that are essential for cancer cell cycle progression and survival. This ionic disruption promotes cell-cycle arrest, particularly at the G_1_ phase, and sensitizes cells to apoptotic cues [14]. Quinidine, closely related to HQ, has been shown to induce apoptosis through the mitochondrial pathway, and similar mechanisms are thought to underlie HQ’s effects [15]. In addition, in silico predictions highlight that both quinidine and HQ can inhibit P-glycoprotein, the multidrug resistance transporter responsible for efflux of many chemotherapeutics, thereby restoring intracellular drug accumulation and potentially overcoming chemoresistance. Early preclinical evidence supports this concept: cinchonine, another cinchona alkaloid, significantly enhanced doxorubicin efficacy in resistant rat colon cancer models by increasing tumor drug uptake and improving survival [16]. While clinical trials with quinidine as a multidrug resistance modulator in breast cancer yielded disappointing results due to limited efficacy at tolerable doses, these studies nevertheless highlight the potential for quinidine derivatives, including HQ, to modulate resistance mechanisms in cancer. Furthermore, computational analyses have suggested that quinidine can bind and inhibit metabolic enzymes such as AKR1B1 and AKR1B10, which are overexpressed in colon carcinoma and contribute to tumor progression [17], thus offering an additional avenue through which HQ might exert antitumor activity.

Despite these promising in vitro findings, the safety and therapeutic efficacy of HQ in vivo remain largely unexplored. While its cardiac side effects are well recognized in clinical practice, little is known about its tolerability in tumor-bearing animals or its ability to suppress cancer progression in vivo. Rat models of colon cancer offer an ideal system to address these questions, as they not only recapitulate key features of human colorectal tumors but also allow simultaneous evaluation of toxicological and therapeutic endpoints. Establishing the safety margins of HQ in rats and determining its capacity to inhibit tumor growth are, therefore, critical steps in assessing its translational potential.

In this study, we sought to address these gaps by evaluating the safety profile of HQ in rats and investigating its antitumor efficacy in a rat model of colon cancer. We hypothesized that HQ, through its ion channel-blocking and regulatory effects on key cellular pathways, would suppress tumor growth while maintaining an acceptable safety profile. To our knowledge, this represents the first systematic in vivo assessment of HQ in colorectal cancer, with implications for its development as a repurposed anticancer therapy.

## 2. Results

### 2.1. Body Weight and General Health Observations

**Figure 1:**
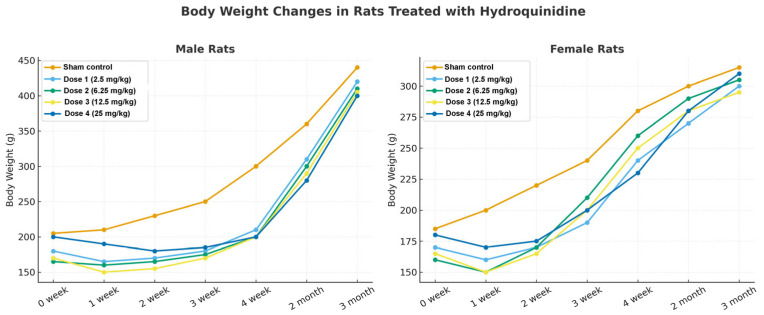
Body weight changes in male (**left**) and female (**right**) Wistar rats treated with HQ for 90 days. Rats were assigned to sham control or treatment groups receiving HQ at 2.5, 6.25, 12.5, or 25 mg/kg. Higher-dose groups initially displayed slower weight gain, but growth normalized over time, indicating adaptation. Values are expressed as mean body weight at each time point (*n* = 6 per group).

The subchronic toxicity of HQ was first assessed in male and female Wistar rats across four dose levels (2.5, 6.25, 12.5, and 25 mg/kg, i.p.), in comparison with vehicle-treated controls (Figure 1). Animals were monitored daily for mortality and morbidity, and body weight was recorded weekly throughout the 90-day study period.

Both male and female rats tolerated HQ at all tested doses without lethality. Body weight analysis revealed that, by the end of the study, sham control animals of both sexes exhibited significantly higher mean body weights compared to HQ-treated groups (Figure 1). A transient reduction in weight gain was observed in rats receiving the higher doses (12.5 and 25 mg/kg) during the initial weeks of treatment. Although body-weight trajectories tended to normalize later in the study, this early pattern may reflect transient treatment-related toxicity or reduced initial tolerability rather than adaptation alone. Additionally, as the study progressed, animals in these higher dose groups resumed normal growth trajectories, suggesting that chronic administration of HQ at elevated doses was eventually well tolerated.

### 2.2. Hematological Findings

Hematological analyses performed at the end of the toxicity study demonstrated dose- and sex-dependent differences (Table 1 and Table 2). In male rats, red blood cell counts (RBC), hemoglobin concentration, hematocrit values (HCT), and fibrinogen levels were significantly reduced in the high-dose groups (12.5 and 25 mg/kg) compared with controls. By contrast, these parameters remained largely unchanged in female rats across all dose groups. This indicates that male rats were more sensitive to HQ-induced hematological alterations, whereas females tolerated the compound more favorably with respect to blood indices. No significant alterations were observed in the remaining hematological parameters, suggesting that HQ treatment was generally well tolerated from a hematological standpoint.

### 2.3. Biochemical Analyses

Biochemical profiling confirmed potential hepatotoxicity at the highest dose tested (Table 3 and Table 4). In the 25 mg/kg HQ group, both male and female rats exhibited significant elevations in serum ALT, AST, and ALP, consistent with liver injury and cholestatic stress. These enzyme increases were accompanied by moderate rises in LDH and GGT, further supporting hepatocellular damage. Importantly, no comparable changes were observed in the lower-dose groups (2.5, 6.25, and 12.5 mg/kg), indicating that hepatotoxicity was dose-dependent and confined to the highest exposure. Although ALT and AST elevations at 25 mg/kg support hepatocellular stress, the concomitant increase in albumin does not indicate impaired hepatic synthetic capacity. This pattern may reflect hemoconcentration, dehydration, acute-phase-related protein redistribution, or compensatory metabolic adaptation. Therefore, the high-dose response is more appropriately interpreted as biochemical evidence of hepatic stress rather than overt hepatic failure.

Renal function markers, including urea and creatinine, remained within physiological ranges across all treatment groups, although a slight upward trend was noted in the 25 mg/kg cohort, suggesting early adaptive responses without overt nephrotoxicity. Similarly, cardiac enzymes (CK-MB, troponin) demonstrated minor fluctuations but did not reach pathologically relevant levels. Lipid metabolism indices, such as cholesterol and triglycerides, showed modest alterations in higher-dose animals, consistent with secondary metabolic stress rather than primary toxicity.

Taken together, these results highlight that HQ was well tolerated at doses up to 12.5 mg/kg, while the 25 mg/kg dose induced clear signs of liver injury. The 6.25 and 12.5 mg/kg doses were selected based on the subchronic tolerability profile rather than pharmacokinetic optimization. The 12.5 mg/kg dose represented the highest dose without overt systemic toxicity, whereas 6.25 mg/kg was included as an intermediate tolerated dose to explore whether HQ-related biological effects could also be detected below the upper tolerated dose. Pharmacokinetic characterization remains an important requirement for future translational studies.

### 2.4. Organ Weights and Histopathological Observations

Postmortem analysis included weighing of major organs and subsequent histopathological assessment (Table 5). Relative organ weights for the brain, heart, kidneys, and gonads did not differ significantly between control and HQ-treated groups in either males or females, indicating that HQ did not exert overt systemic toxicity on these organs. In contrast, liver weights were significantly elevated in the 25 mg/kg group for both sexes, reflecting hepatomegaly. This enlargement of the liver is consistent with the serum biochemical findings, where marked elevations in ALT, AST, and ALP were observed, together supporting the interpretation of hepatocellular stress and injury at the highest dose tested. Notably, no significant alterations in liver size were detected at lower HQ doses (2.5–12.5 mg/kg), in agreement with their more favorable biochemical profile.

### 2.5. Determination of Safe Dose Range

Taken together, these findings indicate that HQ at 2.5, 6.25, and 12.5 mg/kg was well tolerated in both male and female rats, without evidence of systemic toxicity. In contrast, daily treatment at 25 mg/kg induced biochemical and histopathological signs of hepatotoxicity, despite being non-lethal. Therefore, doses up to 12.5 mg/kg were considered safe, and 6.25 and 12.5 mg/kg were selected for subsequent efficacy studies in the colon cancer model.

### 2.6. Histopathological Evaluation of Colon Tissue

**Figure 2:**
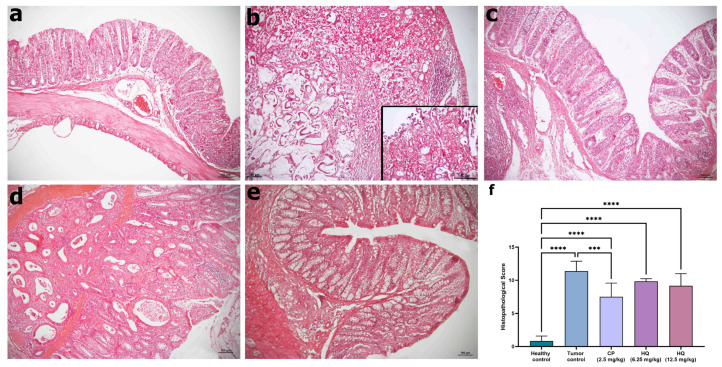
Representative H&E-stained sections of colon tissue. (**a**) Healthy control group; (**b**) tumor control group with detailed carcinomatous structures; (**c**) CP-treated group; (**d**) HQ (6.25 mg/kg) group; (**e**) HQ (12.5 mg/kg) group; (**f**) Histopathological scores of colon tissues from all groups. Scale bars: 50 μm for the inset in panel (**b**) and 100 μm for all other panels. *** *p* < 0.001, **** *p* < 0.0001.

Histological examination of H&E-stained colon sections revealed distinct differences between control and experimental groups (Figure 2). The healthy control group displayed a normal mucosal architecture characterized by orderly arranged glands and crypts with intact epithelial lining (Figure 2a). In contrast, the tumor control (DMH-only) group exhibited profound pathological alterations, including abnormal crypt architecture, increased inflammatory cell infiltration within the lamina propria, epithelial shedding, and a marked reduction in Goblet cell numbers. Additionally, fusion of glands within the muscularis mucosa and submucosa layers was observed, forming carcinomatous structures (Figure 2b).

Treatment with CP partially restored mucosal integrity. Sections from the CP group demonstrated a more regular epithelial arrangement and reduced inflammatory infiltration compared to the cancer group, with a corresponding decrease in carcinomatous lesions (Figure 2c). Rats treated with HQ (6.25 mg/kg) also showed improvement in mucosal and crypt architecture relative to the cancer group, with reduced histological signs of malignancy (Figure 2d). The highest dose of HQ (12.5 mg/kg) produced the most pronounced effect, with epithelial organization approaching near-normal levels, minimal leukocyte infiltration, and reduced carcinomatous features (Figure 2e).

Histopathological scoring supported these observations. The mean histopathological score was 0.82 ± 0.30 in the healthy control group, 11.38 ± 0.53 in the tumor control group, 7.50 ± 0.84 in the CP group, 9.83 ± 0.16 in the 6.25 mg/kg HQ group, and 9.16 ± 0.74 in the 12.5 mg/kg HQ group. Compared with the healthy control group, all experimental groups exhibited significantly higher histopathological scores (**** *p* < 0.0001). Notably, relative to the tumor control group, CP treatment significantly reduced histopathological scores (*** *p* < 0.001), while both HQ-treated groups demonstrated a numerical but less pronounced reduction (Figure 2f).

### 2.7. Caspase-3 Expression as an Apoptosis Marker

**Figure 3:**
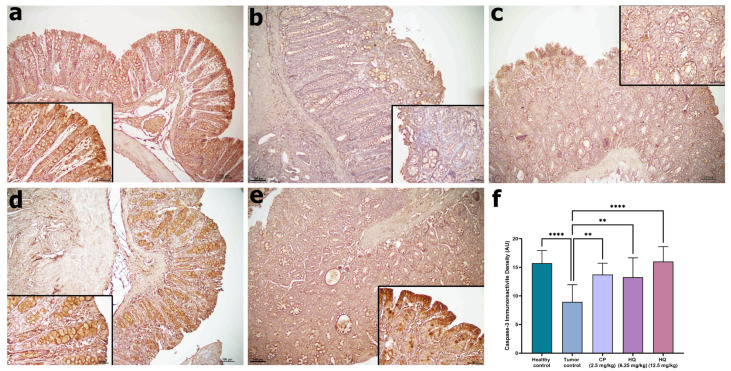
Immunohistochemical analysis of caspase-3 expression in colon tissues. (**a**) Healthy control group; (**b**) tumor control group; (**c**) CP-treated group; (**d**) HQ (6.25 mg/kg) group; (**e**) HQ (12.5 mg/kg) group. Insets show higher magnification views of representative regions. (**f**) Quantification of caspase-3 immunoreactive density in colon tissues across groups. Scale bars: 50 µm for the insets and 100 µm for all other panels. ** *p* < 0.01, **** *p* < 0.0001.

Following H&E staining, caspase-3 immunohistochemistry was performed to assess apoptotic activity across experimental groups (Figure 3). Immunohistochemical staining demonstrated distinct differences in caspase-3 expression. In healthy controls, strong cytoplasmic immunoreactivity for caspase-3 was observed in the colonic epithelium, whereas the tumor control group displayed markedly reduced staining intensity, consistent with impaired apoptotic activity.

CP treatment partially restored caspase-3 expression, as reflected by stronger immunoreactivity compared to the tumor control group. Similarly, HQ administration at both 6.25 and 12.5 mg/kg increased caspase-3 immunoreactive density, with the higher dose producing levels comparable to healthy controls. Quantitative analysis confirmed that caspase-3 expression was significantly lower in the tumor control group compared to healthy controls (**** *p* < 0.0001), while CP (** *p* < 0.01) and HQ treatments (**** p < 0.0001 for 12.5 mg/kg) significantly enhanced caspase-3 expression relative to the tumor control group. Particularly, HQ at 12.5 mg/kg restored caspase-3 expression to control levels, indicating potent pro-apoptotic activity.

### 2.8. IL-6 Expression in Colon Tissue

**Figure 4:**
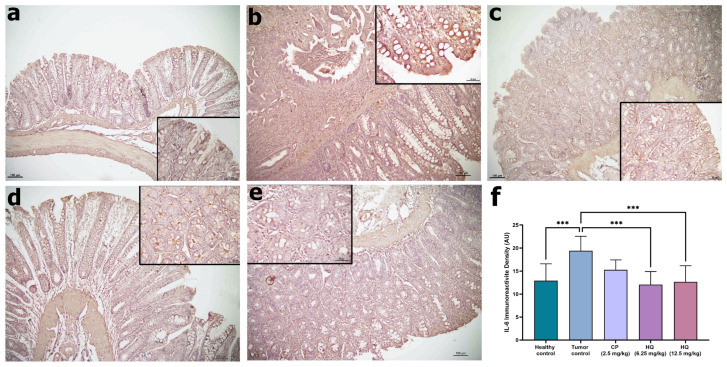
Immunohistochemical analysis of IL-6 expression in colon tissues. (**a**) Healthy control group; (**b**) Tumor control group; (**c**) CP-treated group; (**d**) HQ (6.25 mg/kg) group; (**e**) HQ (12.5 mg/kg) group. Insets show higher magnification views of representative regions. (**f**) Quantification of IL-6 immunoreactive density in colon tissues across groups. Scale bars: 50 µm for the insets and 100 µm for all other panels. *** *p* < 0.001.

To further assess the inflammatory status of the colon tissues, IL-6 immunohistochemistry was performed (Figure 4). Quantitative analysis of immunoreactivity intensity revealed values of 12.91 ± 3.65 in the healthy control group, 19.39 ± 3.17 in the tumor control group, 15.28 ± 2.51 in the CP group, 12.05 ± 2.83 in the 6.25 mg/kg HQ group, and 12.64 ± 3.52 in the 12.5 mg/kg HQ group. In the healthy control group, IL-6 immunoreactivity was low and localized within epithelial cells. In contrast, the tumor control group exhibited strong IL-6 staining, reflecting enhanced inflammatory signaling within the colonic mucosa.

CP treatment reduced IL-6 expression compared to the tumor control group, while HQ at both 6.25 and 12.5 mg/kg markedly suppressed IL-6 staining, approaching levels seen in healthy controls. Quantitative analysis confirmed that IL-6 expression was significantly higher in the tumor control group compared to healthy controls (*** *p* < 0.001), whereas CP and HQ treatments significantly reduced IL-6 immunoreactivity (** *p* < 0.001).

### 2.9. Hydroquinidine Inhibits PI3K/AKT/mTOR Signaling in Colon Cancer

**Figure 5:**
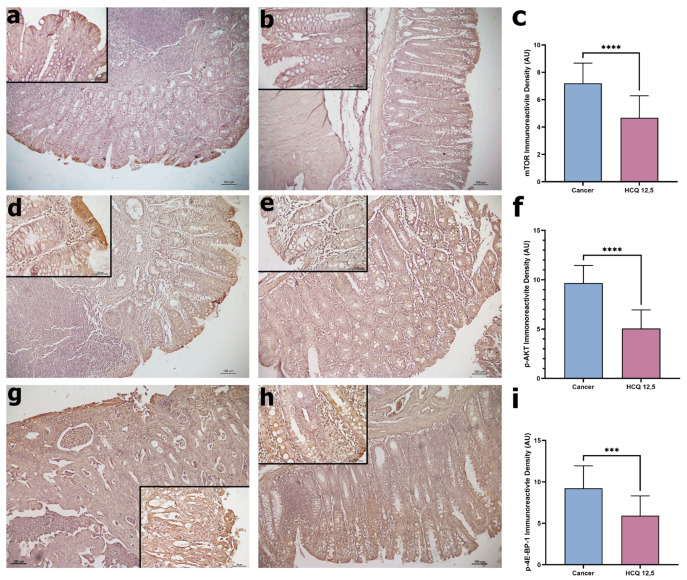
Immunohistochemical analysis of PI3K/AKT/mTOR signaling components in colon cancer tissues. (**a**,**b**) mTOR expression in (**a**) tumor control and (**b**) HQ-treated (**c**) groups with quantification. (**d**,**e**) p-AKT expression in (**d**) tumor control and (**e**) HQ-treated (**f**) groups with quantification. (**g**,**h**) p-4E-BP expression in (**g**) tumor control and (**h**) HQ-treated (**i**) groups with quantification. Data are expressed as mean ± SEM. Statistical significance was determined against the untreated cancer group (**** *p* < 0.0001, *** *p* < 0.001). Scale bars: 50 µm for the insets and 100 µm for all other panels.

To investigate the effect of HQ on the PI3K/AKT/mTOR pathway, we examined mTOR, p-AKT, and p-4E-BP expression in colon tissues using immunohistochemistry (Figure 5). mTOR immunoreactivity was strongly detected in the tumor control group, while HQ treatment (12.5 mg/kg) led to visibly reduced staining. Quantitative analysis confirmed a significant decrease in mTOR expression following HQ treatment (**** *p* < 0.0001). Similarly, p-AKT, a key regulator of mTOR signaling, showed robust expression in tumor control tissues. HQ administration markedly attenuated p-AKT immunoreactivity, with quantitative analysis indicating a significant reduction compared with the tumor control group (**** *p* < 0.0001). Downstream signaling activity was assessed through p-4E-BP, a translational regulator controlled by mTOR. Consistent with the effects on mTOR and p-AKT, HQ treatment significantly reduced p-4E-BP expression relative to tumor control tissues (*** *p* < 0.001). Together, these results demonstrate that HQ treatment suppresses the PI3K/AKT/mTOR pathway in colon cancer tissues, indicating that its antitumor effects may, in part, be mediated through inhibition of this oncogenic signaling cascade.

### 2.10. Hydroquinidine Reverses EMT Phenotype in Colon Tissue

**Figure 6:**
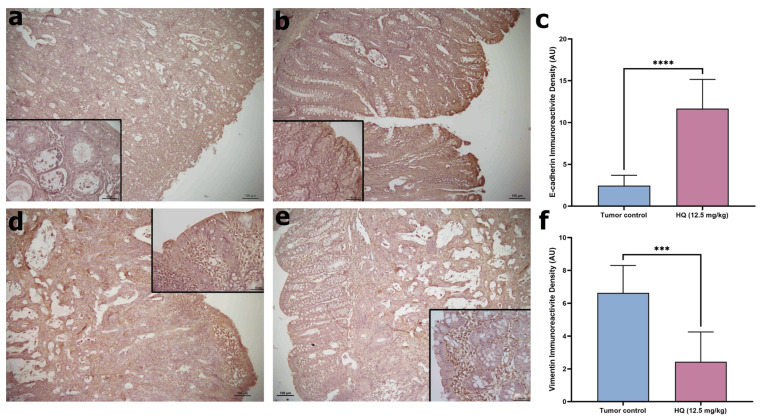
Immunohistochemical assessment of EMT markers in DMH-induced colon cancer. (**a**,**b**) E-cadherin: (**a**) tumor control; (**b**) HQ (12.5 mg/kg). (**c**) groups with quantification. (**d**,**e**) Vimentin: (**d**) tumor control; (**e**) HQ (12.5 mg/kg). (**f**) groups with quantification. Insets show higher-magnification views of representative crypt regions. Quantitative bar plots (immunoreactive density, arbitrary units) accompany the images and are presented as mean ± SEM, with significance tested vs. the untreated cancer group (*** *p* < 0.001, **** *p* < 0.0001). Scale bars: 50 µm for the insets and 100 µm for all other panels.

To explore the impact of HQ on epithelial–mesenchymal transition (EMT) processes, the expression of E-cadherin and vimentin was examined by immunohistochemistry (Figure 6). E-cadherin expression, a hallmark of epithelial integrity and cell–cell adhesion, was assessed by immunohistochemistry (Figure 6a,b). In the healthy control group, E-cadherin was expressed along the basolateral membranes of crypt and surface epithelial cells, supporting normal mucosal architecture. In contrast, tumor control groups are typically characterized by reduced or absent E-cadherin expression. Quantitative analysis revealed an immunoreactivity intensity of 2.45 ± 1.24 in the tumor control group and a markedly elevated 11.68 ± 3.48 in the HQ 12.5 mg/kg group. Compared with tumor control, HQ significantly increased E-cadherin expression (**** *p* < 0.0001), indicating restoration of epithelial adhesion and suppression of malignant dedifferentiation.

Vimentin, a mesenchymal intermediate filament protein associated with invasion and metastasis, was evaluated in colon tissues (Figure 6c,d). Conversely, vimentin expression levels were decreased by HQ. Vimentin immunoreactive density declined from 8.84 ± 3.42 in tumor control to 3.39 ± 1.95 with HQ (*** *p* < 0.001), indicating suppression of mesenchymal features. Together, these reciprocal changes (E-cadherin ↑, vimentin ↓) support an HQ-mediated shift toward an epithelial phenotype.

### 2.11. Hydroquinidine Modulates Inflammation and Oxidative Stress

**Figure 7:**
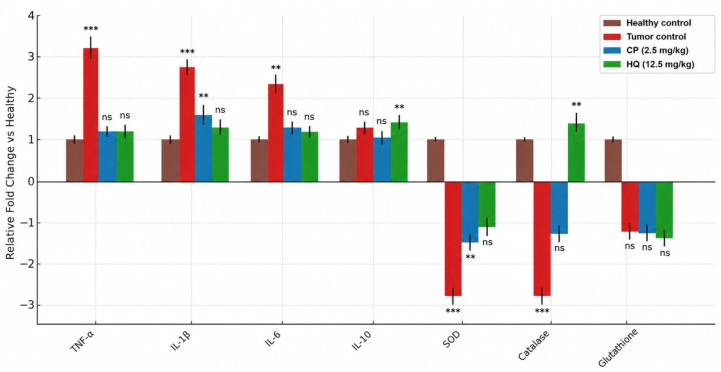
Effects of HQ (12.5 mg/kg) and CP on inflammatory and antioxidant markers in DMH-induced colon cancer rats. Data represent relative fold changes compared to healthy (non-cancer) rats set to 1. Bars represent group means; statistical significance is indicated relative to healthy controls ( ** *p* < 0.01; *** *p* < 0.001; ns, non-significant).

At the last step, ELISA analyses revealed marked alterations in inflammatory and antioxidant markers in the untreated colon cancer group compared with healthy rats (Figure 7). Pro-inflammatory cytokines (TNF-α, IL-1β, IL-6) were significantly elevated in untreated animals, whereas antioxidant enzymes (SOD, catalase) were strongly reduced. Glutathione levels were also lower than in the healthy group. CP treatment partially normalized these parameters, significantly reducing IL-1β and increasing SOD activity. HQ (12.5 mg/kg) showed comparable effects, attenuating IL-10 elevation and restoring catalase activity, while preserving glutathione at levels closer to baseline.

## 3. Discussion

In this study, HQ demonstrated significant protective and antitumor effects in a rat model of DMH-induced colon carcinogenesis. HQ was well-tolerated at doses up to 12.5 mg/kg, with no overt toxicity or weight loss observed, indicating a favorable safety profile at the tested dose range. Importantly, HQ treatment markedly reduced the histopathological damage in colon tissues compared to tumor controls (DMH-only). Treated rats showed preservation of colonic architecture and fewer neoplastic lesions on microscopy, suggesting that HQ mitigated DMH-induced colonic injury. Consistent with these findings, other experimental therapies have likewise improved colonic mucosal integrity and reduced aberrant crypt foci in the DMH model [18,19,20,21]. Our results, therefore, suggest that HQ can suppress colon tumor development and confer tissue-protective effects in this preclinical setting without appreciable toxicity in vivo.

Mechanistically, HQ’s anticancer efficacy in this model appears to be multi-faceted. HQ significantly downregulated IL-6 expression in colon tumor tissue. IL-6 is a pro-inflammatory cytokine implicated in colon carcinogenesis by promoting a chronic inflammatory microenvironment and activating pro-tumorigenic STAT3 signaling [22,23]. The reduction of IL-6 by HQ indicates an attenuation of inflammation, which likely contributed to slower tumor progression. Similar anti-inflammatory effects have been reported with other agents in DMH-induced tumors: for example, raptinal and the natural alkaloid harmol both suppressed IL-6 and TNF-α levels in colon cancer models while activating p53-dependent apoptosis [21,24]. In our study, HQ-treated rats exhibited a restoration of caspase-3 activity in colon tissues, reflecting enhanced apoptosis. DMH carcinogenesis is known to impair apoptotic pathways—prior studies have observed reduced caspase-3 expression in DMH-induced tumors, correlating with uncontrolled cell survival [25,26]. HQ reversed this apoptotic deficit, as evidenced by increased cleaved caspase-3 in treated tumors, indicating that HQ re-engages the intrinsic apoptosis program to eliminate cancer cells. This aligns with reports that HQ and related cinchona alkaloids stimulate the caspase-mediated intrinsic apoptotic pathway in various cancer cell models. Quinine and quinine-derived compounds modulate both drug-resistance mechanisms and intrinsic apoptosis pathways relevant to colon cancer biology. Evidence in the supplied literature shows quinine-containing hybrids produce ROS and mitochondrial damage, while other quinoline/quinone antimalarial analogues or derivatives alter NF-κB, Akt/ERK signaling, and sensitize cells to apoptosis [27,28,29].

In the present study, the PI3K/AKT/mTOR signaling pathway was evaluated using an immunohistochemistry-based approach. Although quantitative techniques such as Western blotting enable global assessment of protein expression levels, immunohistochemistry was considered for visualizing expression patterns and obtaining an overall overview of pathway activity within tissue architecture. Within this context, HQ treatment negatively regulated the PI3K/AKT/mTOR pathway, a master regulator of protein synthesis, metabolism, and proliferation, in the colon cancer model, evidenced by reduced AKT phosphorylation, mTOR levels, and downstream 4EBP1 phosphorylation in HQ-treated tumors versus untreated controls. Notably, the observed suppression of mTOR/AKT signaling provides a plausible mechanistic framework for these effects, as AKT/mTOR is a central pathway driving cell growth and survival in colon cancer and other malignancies [30,31,32]. In colon cancer, where AKT/mTOR is often hyperactive, this may translate, at the preclinical level, to decreased tumor cell growth and increased apoptosis, as observed with HQ therapy. The concomitant reduction in phosphorylated 4EBP1 in HQ-treated tumors is particularly significant, as 4EBP1 is a downstream effector of mTOR that controls cap-dependent translation of pro-growth proteins; its dephosphorylation can halt cancer cell cycle progression [33]. Therefore, the suppression of mTOR, p-AKT, and p-4EBP1 by HQ provides a mechanistic rationale within this experimental model for the inhibited tumor growth seen in treated animals.

By inhibiting AKT and mTOR activity, HQ is likely to shift the balance toward cell-cycle arrest (via diminished 4EBP1-mediated protein synthesis) and apoptosis, impairing tumor expansion. Quinine, a stereoisomer of quinidine, was shown to inhibit AKT activation by preventing its phosphorylation at Thr308/Ser473, which reversed pro-survival signals and induced apoptosis in cancer cells [34]. Mechanistically, quinine and related cinchona alkaloids can bind and inhibit upstream activators of AKT; notably, they competitively bind to the RING domain of TRAF6, an E3 ubiquitin ligase required for AKT activation, thereby blocking AKT’s pro-survival signaling cascade [34]. Moreover, our findings are in harmony with current literature on ion channel modulators. Blocking certain potassium or calcium channels can deactivate AKT/mTOR signaling and enforce tumor-suppressive programs [35,36]. Pharmacological inhibition of hERG channels has been shown to block this signaling cascade, leading to reduced tumor growth and increased apoptosis in colon cancer models [37]. Likewise, in another study, modulation of ion channels led to a significant increase in cytosolic calcium in colon cancer cells, which in turn induced apoptosis in HT29 and HCT116 cell lines [38].

HQ treatment also modulated key protein markers of EMT, a process linked to tumor invasiveness and metastasis. We observed upregulation of E-cadherin and concomitant downregulation of vimentin in HQ-treated colon tumors, relative to untreated tumor controls. E-cadherin is a cell–cell adhesion molecule typically lost during EMT, while vimentin is a mesenchymal cytoskeletal protein that is gained as tumors become more invasive [39,40]. The inverse expression changes of these markers are strongly associated with reduced metastatic potential. In colorectal cancer patients, lower E-cadherin and higher vimentin levels correlate with higher tumor grade, lymphovascular invasion, and metastasis [41,42]. By restoring E-cadherin expression and suppressing vimentin, HQ likely helped maintain an epithelial phenotype in tumor cells and impeded the EMT process. This suggests that HQ not only inhibits primary tumor growth in the presented model but may also reduce the likelihood of invasion and metastasis. Supporting this interpretation, interventions that prevent the EMT-associated E-cadherin loss (or vimentin rise) have been shown to limit colorectal cancer aggressiveness [42,43]. Thus, HQ’s ability to favorably alter these molecular markers in our study points to a potential anti-metastatic action, consistent with previous studies reporting anti-migratory effects of HQ in various cancers [10,11,12].

Our findings align with the growing body of literature highlighting the broad in vitro anticancer properties of HQ. Recent studies have shown that HQ exerts potent antitumor effects in multiple malignancies, highlighting its promise as a repurposed anticancer agent. Yavuz et al. [11] reported that HQ significantly inhibited the proliferation and clonogenic survival of breast (MCF-7) and ovarian (SKOV-3) cancer cells, inducing cell-cycle arrest and robust apoptosis. Proteomic analyses in that study revealed downregulation of cell cycle drivers and upregulation of apoptotic pathways in HQ-treated cells, mechanistically underpinning its antiproliferative activity [11]. In non-small cell lung cancer, HQ similarly reduced colony formation and tumorigenicity of A549 cells, and it markedly impeded cancer cell migration in vitro [13]. Gene expression profiling confirmed that HQ downregulated genes involved in cell division and survival while upregulating those promoting cell cycle arrest and apoptosis in lung cancer cells [13]. Notably, HQ has also demonstrated efficacy against glioblastoma, one of the most aggressive and therapy-resistant cancers. In both temozolomide-sensitive and temozolomide-resistant glioblastoma cell lines, HQ treatment increased apoptotic cell death and decreased proliferation rates, accompanied by alterations in gene networks governing cell cycle progression and cell survival [44]. These consistent outcomes across cancer types (colon, breast, ovarian, lung, and brain tumors) indicate that HQ’s anti-neoplastic effects are not confined to a single tissue but rather reflect a multi-modal mechanism of action that is broadly effective. Indeed, HQ is a multi-ion channel blocker, and there is evidence that interfering with ion channel activity can disrupt cancer cell homeostasis, invasiveness, and metabolic adaptation [45,46,47]. The collective evidence strongly supports HQ as a promising preclinical candidate for drug repurposing in oncology, with studies unanimously reporting its ability to curb cancer cell growth, trigger apoptosis, and even diminish migratory/invasive properties.

Furthermore, our data indicate that HQ treatment mitigates the inflammatory response and restores redox balance in the colon cancer model. The suppression of TNF-α and IL-6, together with the recovery of catalase activity, suggests that HQ exerts both anti-inflammatory and antioxidant effects. Such dual activity is consistent with the drug’s broader reported anticancer mechanisms, including modulation of oxidative stress pathways. Although CP displayed stronger effects on IL-1β and SOD activity, HQ’s ability to normalize multiple parameters without overt toxicity highlights its potential as a supportive or alternative therapeutic candidate. Therefore, our findings establish the antitumor and safety profile of HQ in a preclinical colon cancer model.

Despite these encouraging findings, several limitations should be acknowledged. Although HQ improved histopathological features and modulated inflammation-, apoptosis-, EMT-, oxidative stress-, and PI3K/AKT/mTOR pathway-associated markers, the present study did not assess formal antitumor efficacy endpoints such as macroscopic tumor burden, tumor multiplicity, tumor volume, metastatic spread, survival, or proliferation markers such as Ki-67 or PCNA. Therefore, the findings should be interpreted as evidence of tumor-associated histopathological and molecular modulation rather than definitive proof of reduced tumor growth or improved survival. In addition, the study was limited to the DMH-induced rat model, which does not fully reproduce the molecular heterogeneity, metastatic progression, or therapeutic complexity of human colorectal cancer. Dose selection was based on subchronic tolerability rather than pharmacokinetic or exposure–response optimization, and future pharmacokinetic, tissue distribution, and dose–response studies will be required to better define the therapeutic window of HQ. Moreover, the precise molecular targets of HQ in vivo remain unclear. Since ion-channel expression or activity was not directly evaluated in the present in vivo model, the potential contribution of HQ-mediated potassium-channel blockade to the observed histopathological and molecular changes should be interpreted as a pharmacological hypothesis rather than a demonstrated mechanism. Future studies incorporating hERG1/Kv channel expression analyses and functional assays will be required to establish this link. Finally, although IHC provided valuable spatial information, complementary quantitative and functional approaches, including Western blotting, RT-qPCR, proteomics, and pathway-specific validation assays, are needed to strengthen the mechanistic interpretation. Future studies should also address sex-dependent hematological differences and evaluate HQ in combination with standard therapies or optimized delivery systems such as nanoencapsulation [10].

## 4. Materials and Methods

### 4.1. Animals and Housing

All animal experiments were carried out in accordance with the ARRIVE guidelines and recognized international standards for the care and use of laboratory animals and conducted at the Muğla Sıtkı Koçman University Experimental Animal Research Center under controlled conditions. The protocol was approved by the institutional ethics committee and carried out in compliance with international guidelines for laboratory animal care (ID: NO. 36/22; 18 August 2022). Adult Wistar albino rats were used for all in vivo studies. Rats were housed in standard cages in a temperature- (23 ± 2 °C) and humidity-controlled environment (50–55% relative humidity) with a 12 h light/dark cycle. Standard rat chow and water were provided ad libitum. Prior to group assignment, animals were acclimatized and randomized such that mean body weights differed by no more than ±25% across groups. All treatments and measurements were done by the same researcher. After each treatment, the cages were returned to their designated positions to maintain environmental consistency. The veterinary technician who transported the animals and the researchers responsible for administering the treatments were aware of the group allocations. Exclusion criteria for animals in the study included weight loss exceeding 15% of body weight, behavioral disorders, inability to properly access food and water, markedly reduced responsiveness to stimuli, and euthanasia deemed necessary by the veterinarian (for humane reasons). Additionally, if more than 20% of the animals in the project died for any reason during the experiment, those animals were also excluded from the study. No animals met the predefined exclusion criteria; therefore, all animals were included in both the experimental procedures and the final data analyses.

### 4.2. Dose Selection and Subchronic Toxicity Study

The safety profile of HQ was evaluated according to OECD guidelines for acute and repeated-dose toxicity [48]. Preliminary acute toxicity tests established LD_50_ values of approximately 140 mg/kg (intraperitoneal, i.p.) and 324 mg/kg (oral) for HQ, classifying it as moderately toxic (Hodge–Sterner toxicity Category 3). The dose selection for HQ, a Class I antiarrhythmic, was guided by its Category 3 toxicity to ensure a sufficient safety margin and to simulate realistic exposure from repeated dosing. Accordingly, four doses (2.5, 6.25, 12.5, and 25 mg/kg) were selected for the 90-day subchronic toxicity study. These doses were all well below the experimentally determined LD_50_ values and were chosen to span low to moderately high exposure levels within the expected safety window of Class I antiarrhythmic compounds, enabling the evaluation of dose-dependent safety and potential toxicity.

Cinchona alkaloids, including hydroquinidine, exhibit low and variable oral bioavailability in rodents due to extensive first-pass metabolism and gastrointestinal degradation [49]. Therefore, i.p. administration is preferred to minimize inter-animal variability in drug absorption and to enable reliable assessment of pharmacological and toxicological effects. Regarding the translational context, rat efficacy doses were subsequently converted to human equivalent doses using body surface area-based allometric scaling [50]. The resulting estimates corresponded to dose levels substantially lower than those used clinically for antiarrhythmic indications (600–900 mg per day to manage Brugada syndrome or refractory arrhythmias [51]), supporting the selection of these doses for preclinical evaluation while maintaining an adequate safety margin.

**Figure 8:**
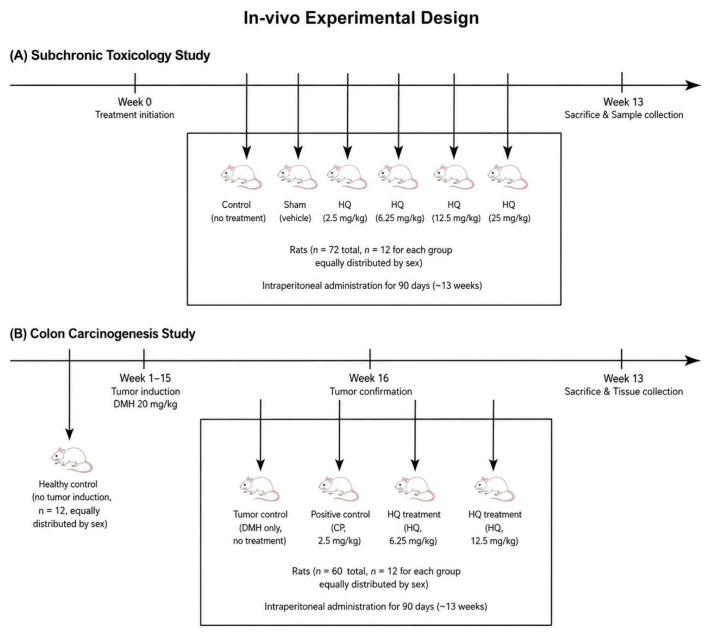
In vivo experimental design of the study. (**A**) Subchronic toxicology study. Seventy–two rats, equally distributed by sex, were randomly assigned to six experimental groups. At the end of the treatment period, animals were sacrificed, and biological samples were collected for toxicological and biochemical evaluations. (**B**) Colon carcinogenesis study. Twelve rats served as healthy controls and did not receive dimethylhydrazine (DMH). Sixty rats were used for the colon cancer model and received DMH (20 mg/kg) for 15 weeks to induce colon carcinogenesis. Following tumor confirmation at week 16, animals were allocated to the four groups. Treatments were administered for 90 days, after which animals were sacrificed, and colon tissues were collected for histopathological, immunohistochemical, and biochemical analyses.

A total of 72 rats (36 female, 36 male) were randomly divided into six groups according to the applied Power analysis (Power = 0.82, f = 0.40, alpha = 0.05) (*n* = 12 per group, 6 ♀ + 6 ♂ each): a control group (no treatment), a sham vehicle group, and four HQ dose groups (Figure 8). HQ was dissolved in DMSO (Neofroxx, Einhausen, Germany, Catalog no: 1264ML100) by ultrasonication, diluted to the tested concentrations in corn oil, and administered intraperitoneally at a volume of 0.5 mL once daily for 90 days. Control animals received no injections, and sham animals received volume-matched vehicle injections on the same schedule.

### 4.3. Clinical Observations and Sample Collection

All animals were observed twice daily for mortality or any signs of morbidity throughout the study. Detailed clinical examinations were performed, noting any changes in skin and mucous membranes, signs of autonomic dysfunction (e.g., piloerection, altered respiration), behavioral abnormalities, or impaired motor function. Body weights and food intake were recorded weekly. On day 90 of the study, blood samples were collected from each animal under light anesthesia. Samples were drawn via cardiac puncture or tail vein (as appropriate) into both heparinized tubes (for plasma) and plain tubes (for serum). At the end of 90 days, all surviving rats were humanely euthanized, and a full necropsy was performed. Major organs (brain, liver, kidneys, heart, and gonads) were excised and examined grossly.

### 4.4. Hematological and Biochemical Analysis

Complete blood counts were measured from anticoagulated whole blood using a Sysmex XS-1000i hematology analyzer (Sysmex Corp., Kobe, Japan). Parameters assessed included total and differential leukocyte counts, erythrocyte indices, hemoglobin, hematocrit, and platelet count. Serum biochemical parameters were analyzed with a Siemens Centaur CP automated analyzer. The following clinical chemistry endpoints were measured to evaluate organ function and systemic toxicity: For liver enzymes: Alanine aminotransferase (ALT), Aspartate aminotransferase (AST), Gamma-glutamyl transferase (GGT), Lactate dehydrogenase (LDH); for renal function: Blood urea nitrogen (urea) and creatinine; for metabolic status: Glucose, total protein, albumin, total cholesterol, and for cardiac injury markers: Creatine kinase-MB (CK-MB) and cardiac troponin. All assays were performed according to the manufacturers’ protocols and calibrated quality controls.

### 4.5. Colon Carcinogenesis Model and Treatment

An experimental colon cancer model was established using the chemical carcinogen 1,2-dimethylhydrazine (DMH). This model is a well-established proxy for sporadic colorectal cancer in humans [52]. Briefly, 60 young adult Wistar rats (30 male, 30 female, ~8 weeks old) received weekly subcutaneous injections of DMH (20 mg/kg body weight) for 15 consecutive weeks to induce colonic tumor formation [53] (Figure 8). Repeated exposure to DMH is known to produce colon tumors with histopathological features similar to human colon adenocarcinoma. During the induction period, a separate group of rats (negative control for carcinogenesis, *n* = 12, 6 ♂ + 6 ♀) was maintained without exposure to DMH. Tumor development was considered successful once the average tumor volume reached 25–50 mm^3^, as assessed by palpation and confirmed at necropsy at week 16. The tumor-bearing rats were then randomized into four treatment groups (*n* = 12 per group, with equal sex distribution): the tumor control group received no anticancer treatment after DMH. Positive control (chemotherapy) group animals were treated with cisplatin (CP, 2.5 mg/kg, i.p.) every 3 weeks. For HQ groups, animals were treated with HQ at the two highest safe doses (6.25 or 12.5 mg/kg, i.p. daily).

Therapies commenced in week 16 after tumor confirmation and continued for up to 90 days. CP was administered on a tri-weekly schedule to mimic a clinical chemotherapy regimen, while HQ was given daily. All colon tumor model rats were monitored throughout the treatment phase for general health, and body weights were measured weekly to assess treatment tolerability. At study termination, rats were euthanized and examined for tumor burden. Colonic tumors were carefully dissected out, counted, and measured as described below. Tumor tissues were then fixed in 10% buffered formalin for histological and immunohistochemical evaluation, or snap-frozen for biochemical assays. Because macroscopic tumor burden parameters such as tumor multiplicity, tumor volume, and survival were not quantitatively analyzed in the present report, the findings should be interpreted as evidence of tumor-associated histopathological and molecular modulation rather than definitive proof of reduced tumor burden.

### 4.6. Histopathological Evaluations of Colon Tissue

Colon samples were fixed in 10% neutral buffered formalin (NBF) for 24 h and subsequently washed under running tap water. The tissues were dehydrated through a graded ethanol series (70%, 95%, and 100%) for 1 h, cleared in xylene for 40 min, and embedded in paraffin. Paraffin blocks were sectioned at 5 µm thickness using a rotary microtome. The sections were stained with hematoxylin–eosin (H&E) for histological evaluation [54]. For morphological evaluation, the colon sections were incubated in xylene for 30 min, followed by rehydration through a descending ethanol series (100%, 95%, and 70%) for 10 min each. The sections were then immersed in Mayer’s hematoxylin solution for 7 min and rinsed under tap water for 10 min. Subsequently, the slides were stained with 1% eosin for 3 min, rinsed in distilled water, and dehydrated sequentially in an ethanol series. Finally, the sections were cleared in xylene and mounted with Bio-Mount. Histopathological scoring was performed semi-quantitatively by evaluating mucosal architecture, crypt distortion, epithelial damage, inflammatory cell infiltration, goblet cell depletion, dysplasia, and carcinomatous lesion severity (0: none, 1: mild, 2: moderate, 3: marked, 4: severe) with a maximum total score of 16 per section [55]. Scoring was conducted blindly by two independent researchers to ensure objectivity and reproducibility. To standardize field sampling, ten random fields at 400× magnification per section were evaluated for each animal, and the mean score was calculated.

### 4.7. Immunohistochemistry of Cancer-Related Markers

The colon sections were incubated in xylene for 30 min, followed by rehydration through a descending ethanol series (100%, 95%, and 70%) for 10 min each. Endogenous peroxidase activity was blocked using 3% hydrogen peroxide (H_2_O_2_) for 20 min. For antigen retrieval, the slides were heated in a microwave oven (200 W, 20 min) in citrate buffer (pH 6.0) within a plastic container, then cooled at room temperature for 20 min. After cooling, the sections were rinsed in phosphate-buffered saline (PBS). Immunohistochemical staining was performed using a commercial detection kit (Abcam, Cambridge, UK, Catalog no: ab93677) according to the manufacturer’s protocol with minor modifications. The slides were incubated in blocking solution for 15 min. The colon sections were subsequently incubated overnight at 4 °C in humidified chamber with following antibodies: anti-caspase-3 (1:100, Elabscience, Wuhan, Hubei, China, Catalog no: E-AB-63510), anti-IL-6 (1:250, Abcam, Catalog no: ab9324), anti-E-cadherin (1:1000, Proteintech, Chicago, IL, USA, Catalog no: 20874-1-AP), anti-vimentin (1:400, Cell Signaling, Danvers, MA, USA, Catalog no: D21H3), mTOR (1:200, Cell Signaling, Catalog no: 7C10), p-AKT (1:100, Cell Signaling, Danvers, MA, USA, Catalog no: 4060), and p-4E-BP-1 (1:2000, Cell Signaling, Catalog no: 236B4). On the following day, after rinsing with PBS, the sections were incubated with a biotinylated secondary antibody for 10 min at room temperature, followed by streptavidin-peroxidase for 10 min. The slides were then rinsed three times with PBS and visualized using 3,3′-diaminobenzidine (DAB) substrate solution (Abcam, Catalog no: ab64238) for 1 min. Counterstaining was performed with Mayer’s hematoxylin for 7 min, followed by running tap water for 10 min. The sections were dehydrated through the graded ethanol series (70%, 95%, and 100%), cleared in xylene for 1 min, and mounted with Bio-Mount. The immunoreactivity intensity was assessed in ten randomly selected fields per section at 400× magnification and quantified using Image J software (1.44 software, National Institutes of Health, Bethesda, MD, USA). For each section, representative non-overlapping regions of interest were selected while avoiding artefactual areas. Color deconvolution was applied to separate DAB staining, and the background signal was corrected using unstained or weakly stained tissue regions. A constant threshold was then applied across all groups, and immunoreactive density was expressed in arbitrary units. The investigator performing quantification was blinded to group allocation.

For negative controls, sections were processed using the same staining protocol with the omission of the primary antibody. The antibodies used were selected based on manufacturer validation for immunohistochemistry and prior use in tissue-based applications. Staining patterns were evaluated together with tissue morphology and negative control sections to minimize the interpretation of non-specific background signals.

### 4.8. Tumor Tissue Homogenate Analyses (Cytokines and Antioxidants)

Biochemical analyses were performed on tumor tissue to investigate the inflammatory and oxidative stress status. Portions of fresh tumor tissue were weighed and homogenized in ice-cold PBS (pH 7.4) using a glass homogenizer. Homogenates were prepared at ~10% *w*/*v* (e.g., 0.1 g tissue per 1 mL PBS) and then centrifuged at 10,000× *g* for 15 min at 4 °C. The supernatants were collected and stored at −80 °C until assay. The biomarkers Tumor necrosis factor-alpha (TNF-α), interleukin-1 beta (IL-1β), interleukin-6 (IL-6), Interleukin-10 (IL-10), Superoxide dismutase (SOD), catalase (CAT), and Reduced glutathione (GSH)) were measured in the tumor supernatants, using commercially available ELISA kits (Elabscience, Wuhan, Hubei, China). All assays were performed in duplicate and according to the kit instructions.

### 4.9. Statistical Analyses

All data were analyzed using R statistical software (version 4.2.3) with the built-in stats package. Prior to significance testing, datasets were checked for normal distribution using the Shapiro–Wilk test, and homogeneity of variance was evaluated using Levene’s or Brown–Forsythe test where appropriate. Continuous variables are presented as mean ± standard error of the mean (SEM) for each experimental group. For endpoints that met assumptions of normality and homogeneity of variances, one-way analysis of variance (ANOVA) was applied, followed by Tukey’s post hoc test to evaluate pairwise group differences. For non-normally distributed data, a non-parametric Kruskal–Wallis test was used, followed by Dunn’s multiple comparison test for post-hoc analysis. Statistical significance was accepted at *p* < 0.05. Significant differences between experimental groups are indicated in the figures and tables with appropriate symbols or letters.

## 5. Conclusions

This study provides comprehensive preclinical evidence that HQ, an established antiarrhythmic agent, exerts both safe and effective anticancer activity in a chemically induced rat model of colorectal cancer. Toxicological analyses confirmed that HQ was well tolerated at doses up to 12.5 mg/kg, whereas 25 mg/kg induced hepatotoxicity without lethality. Importantly, therapeutic dosing with HQ attenuated DMH-induced carcinogenesis, ameliorated histopathological damage, and significantly modulated key molecular markers. HQ suppressed IL-6 expression, enhanced caspase-3-mediated apoptosis, and improved E-cadherin expression, while reducing vimentin levels, thereby supporting mucosal integrity and inhibiting epithelial–mesenchymal transition. Collectively, these findings position HQ as a promising preclinical candidate for drug repurposing in colorectal cancer. Its dual demonstration of tolerability and tumor-suppressive effects underscores its translational potential. Future studies should aim to validate these findings in additional in vivo models, explore combination strategies with standard chemotherapeutics, and elucidate the mechanistic basis of EMT marker modulation. Ultimately, HQ may contribute to the development of novel therapeutic strategies targeting both tumor growth and microenvironmental regulation in colorectal cancer, pending further preclinical and translational investigations.

## Figures and Tables

**Table 1:** Hematological parameters of male Wistar rats following 90-day HQ administration (mean ± SEM, ** *p* < 0.01, *n* = 6).

Parameter	Control	Dose 1 (2.5 mg/kg)	Dose 2 (6.25 mg/kg)	Dose 3 (12.5 mg/kg)	Dose 4 (25 mg/kg)
WBC (10^3^/mm^3^)	9.65 ± 2.39	9.56 ± 1.77	10.3 ± 2.93	8.94 ± 2.56	7.71 ± 2.43
RBC (10^6^/mm^3^)	7.89 ± 0.18	7.65 ± 0.32	7.23 ± 0.33 **	7.18 ± 0.27 **	7.16 ± 0.46 **
Hemoglobin (g/dL)	15.2 ± 0.4	14.8 ± 0.5	13.9 ± 0.8 **	13.6 ± 0.3 **	13.2 ± 0.3 **
HCT (%)	45.6 ± 1.8	44.6 ± 1.5	42.5 ± 2.4	41.9 ± 1.2 **	40.7 ± 0.9 **
MCV (µm^3^)	57.8 ± 1.9	58.3 ± 1.5	58.7 ± 1.2	58.3 ± 1.7	57.0 ± 2.7
MCH (pg)	19.3 ± 0.7	19.4 ± 0.6	19.3 ± 0.4	19.0 ± 0.9	18.4 ± 0.9
MCHC (%)	33.4 ± 0.7	33.2 ± 0.5	32.8 ± 0.2	32.5 ± 0.7	32.3 ± 0.3
Reticulocytes (%)	2.8 ± 0.3	3.3 ± 0.4	3.2 ± 0.3	3.9 ± 0.5	3.2 ± 1.0
Platelets (10^3^/mm^3^)	1202 ± 75	1265 ± 107	1280 ± 116	1572 ± 430	1639 ± 227
Fibrinogen (mg/dL)	249 ± 13	224 ± 8	189 ± 15 **	198 ± 21 **	193 ± 20 **

**Table 2:** Hematological parameters of female Wistar rats following 90-day HQ administration (mean ± SEM, *n* = 6).

Parameter	Control	Dose 1 (2.5 mg/kg)	Dose 2 (6.25 mg/kg)	Dose 3 (12.5 mg/kg)	Dose 4 (25 mg/kg)
WBC (10^3^/mm^3^)	6.56 ± 2.14	7.01 ± 3.5	5.97 ± 1.99	6.31 ± 1.33	6.47 ± 1.83
RBC (10^6^/mm^3^)	7.81 ± 0.38	7.62 ± 0.61	7.79 ± 0.22	7.46 ± 0.30	7.49 ± 0.30
Hemoglobin (g/dL)	15.1 ± 0.9	14.9 ± 1.3	15.2 ± 0.4	14.8 ± 0.7	14.1 ± 0.6
HCT (%)	43.7 ± 1.7	43.5 ± 3.1	44.0 ± 1.3	43.1 ± 1.8	41.6 ± 1.6
MCV (µm^3^)	56.0 ± 1.1	57.1 ± 1.6	56.4 ± 0.8	57.7 ± 1.4	55.6 ± 1.0
MCH (pg)	19.3 ± 0.4	19.6 ± 0.6	19.5 ± 0.4	19.8 ± 0.5	18.9 ± 0.4
MCHC (%)	34.5 ± 0.8	34.3 ± 0.8	34.5 ± 0.4	34.4 ± 0.3	34.0 ± 0.4
Reticulocytes (%)	2.1 ± 0.4	3.5 ± 1.7	2.6 ± 0.4	2.5 ± 0.2	2.4 ± 0.3
Platelets (10^3^/mm^3^)	1295 ± 118	1360 ± 155	1367 ± 79	1368 ± 138	1350 ± 194
Fibrinogen (mg/dL)	193 ± 11	222 ± 46	186 ± 9	184 ± 29	155 ± 10

**Table 3:** Biochemical parameters of male Wistar rats following 90-day HQ administration (mean ± SEM, * *p* < 0.05, ** *p* < 0.01*, n* = 6).

Parameter	Control	Dose 1 (2.5 mg/kg)	Dose 2 (6.25 mg/kg)	Dose 3 (12.5 mg/kg)	Dose 4 (25 mg/kg)
Total protein (mg/dL)	5840 ± 340	5520 ± 100	5550 ± 240	5720 ± 220	5860 ± 400
Albumin (mg/dL)	3780 ± 220	3900 ± 170	4060 ± 200	4430 ± 180 *	4400 ± 410 **
Glucose (mg/dL)	122 ± 13	132 ± 15	170 ± 18 **	170 ± 10 **	156 ± 16 **
Total cholesterol (mg/dL)	59 ± 11	46 ± 9	45 ± 4	49 ± 13	52 ± 20
Triglycerides (mg/dL)	25.5 ± 8.4	24.3 ± 4.5	34.5 ± 7.1	44.8 ± 20.9	45.8 ± 12.5
BUN (mg/dL)	13.0 ± 2.5	12.9 ± 0.5	15.5 ± 1.7	15.8 ± 1.3	17.2 ± 2.4 **
Urea (mg/dL)	55.1 ± 2.6	53.0 ± 2.3	61.0 ± 3.0	56.0 ± 2.7	66.8 ± 1.6 **
AST (U/L)	72 ± 7	71 ± 11	75 ± 5	83 ± 22	115 ± 16 **
ALT (U/L)	30 ± 5	28 ± 4	32 ± 3	48 ± 10 *	53 ± 17 *

**Table 4:** Biochemical parameters of female Wistar rats following 90-day HQ administration (mean ± SEM, * *p* < 0.05, ** *p* < 0.01, *n* = 6).

Parameter	Control	Dose 1 (2.5 mg/kg)	Dose 2 (6.25 mg/kg)	Dose 3 (12.5 mg/kg)	Dose 4 (25 mg/kg)
Total protein (mg/dL)	5680 ± 140	5610 ± 180	5530 ± 190	5930 ± 330	5850 ± 190
Albumin (mg/dL)	3810 ± 230	3670 ± 430	3720 ± 120	4120 ± 140	4210 ± 180
Glucose (mg/dL)	110 ± 15	120 ± 20	114 ± 16	127 ± 22	151 ± 8 **
Total cholesterol (mg/dL)	49 ± 10	59 ± 5	50 ± 7	54 ± 6	84 ± 16 **
Triglycerides (mg/dL)	12.3 ± 5.6	12.1 ± 2.6	8.8 ± 3.7	12.2 ± 1.1	31.9 ± 4.8 **
BUN (mg/dL)	16.1 ± 4.3	15.5 ± 1.5	16.6 ± 3.8	15.8 ± 2.4	16.9 ± 1.3
Urea (mg/dL)	57.4 ± 3.9	59.7 ± 3.2	60.1 ± 2.9	58.1 ± 2.2	68.4 ± 2.7 *
AST (U/L)	68 ± 5	69 ± 11	66 ± 7	68 ± 9	86 ± 12 *
ALT (U/L)	21 ± 2	22 ± 4	23 ± 3	27 ± 4	33 ± 6 **

**Table 5:** Organ weights of Wistar rats following 90-day HQ administration (mean ± SEM, ** *p* < 0.01, *n* = 6).

Sex/Organ	Control	Dose 1 (2.5 mg/kg)	Dose 2 (6.25 mg/kg)	Dose 3 (12.5 mg/kg)	Dose 4 (25 mg/kg)
**Male**					
Brain (g)	2.02 ± 0.08	2.03 ± 0.07	2.12 ± 0.06	2.07 ± 0.10	2.10 ± 0.10
Heart (g)	1.09 ± 0.09	1.10 ± 0.11	1.20 ± 0.10	1.18 ± 0.07	1.28 ± 0.16
Liver (g)	9.40 ± 0.58	11.65 ± 1.90	11.8 ± 1.64	17.11 ± 3.46 **	20.61 ± 3.36 **
Kidney (g)	1.70 ± 0.14	1.61 ± 0.08	1.71 ± 0.09	1.72 ± 0.11	1.87 ± 0.19
Testis (g)	2.90 ± 0.16	2.84 ± 0.12	3.13 ± 0.11	2.91 ± 0.15	3.07 ± 0.18
**Female**					
Brain (g)	1.94 ± 0.10	1.92 ± 0.08	1.95 ± 0.07	1.94 ± 0.05	1.99 ± 0.02
Heart (g)	0.75 ± 0.07	0.77 ± 0.03	0.75 ± 0.02	0.79 ± 0.04	0.87 ± 0.06
Liver (g)	6.39 ± 0.87	6.84 ± 0.63	6.73 ± 0.26	8.85 ± 0.99 **	12.43 ± 0.89 **
Kidney (g)	1.70 ± 0.14	1.61 ± 0.08	1.71 ± 0.09	1.72 ± 0.11	1.87 ± 0.19
Ovary (g)	0.087 ± 0.022	0.096 ± 0.018	0.082 ± 0.011	0.097 ± 0.009	0.089 ± 0.018

## Data Availability

The original contributions presented in this study are included in the article. Further inquiries can be directed to the corresponding authors.

## Abbreviations

The following abbreviations are used in this manuscript:

HQ	Hydroquinidine
OECD	Organisation for Economic Co-operation and Development
DMH	1,2-Dimethylhydrazine
IL-6	Interleukin-6
IL-1ß	Interleukin-1 beta
IL-10	Interleukin-10
TNF-α	Tumor Necrosis Factor-alpha
mTOR	Mechanistic Target of Rapamycin
PI3K	Phosphoinositide 3-kinase
AKT	Protein Kinase B
p-AKT	Phosphorylated AKT
p-4E-BP1	Phosphorylated Eukaryotic Translation Initiation Factor 4E-Binding Protein 1
EMT	Epithelial–Mesenchymal Transition
hERG (Kv 11.1)	Human Ether-à-go-go-Related Gene Potassium Channel 1
AKR1B1	Aldo-Keto Reductase Family 1 Member B1
AKR1B10	Aldo-Keto Reductase Family 1 Member B10
RBC	Red Blood Cells
WBC	White Blood Cells
HCT	Hematocrit
MCV	Mean Corpuscular Volume
MCH	Mean Corpuscular Hemoglobin
MCHC	Mean Corpuscular Hemoglobin Concentration
ALT	Alanine Aminotransferase
AST	Aspartate Aminotransferase
ALP	Alkaline Phosphatase
LDH	Lactate Dehydrogenase
GGT	Gamma-Glutamyl Transferase
BUN	Blood Urea Nitrogen
CK-MB	Creatine Kinase-MB
H&E	Hematoxylin and Eosin
PBS	Phosphate-Buffered Saline
DMSO	Dimethyl Sulfoxide
NBF	Neutral Buffered Formalin
ELISA	Enzyme-Linked Immunosorbent Assay
SOD	Superoxide Dismutase
CAT	Catalase
GSH	Reduced Glutathione
SEM	Standard Error of the Mean
ANOVA	Analysis of Variance
CP	Cisplatin
